# 

**Published:** 1897-11-20

**Authors:** 


					THE HOSPITAL. ABSOLUTELY PURE, therefore BEST. Not. 20th, 1897. /)
CADBURY'S ^ ww ** CADBURY'S
GOCOA rlr Ir55! JST^ COCOA
" The standard of highest purity at present ?   AT ? ? T
attainable."?Lancet. ^ NO ALKALIES USED.
Mi
r/?W/CH HOSPl.TAt
No. 582: Vol. XXIII. [S^per!]. Saturday,
Nov. 20tii, 1897.
[SubscriptionTemB.] pRICI; TWOPENCE.

				

